# Political economy and population health: is Australia exceptional?

**DOI:** 10.1186/1743-8462-3-6

**Published:** 2006-06-01

**Authors:** Anne-marie Boxall, Stephanie D Short

**Affiliations:** 1 Australian Health Policy Institute, Ground Floor, Victor Coppleson Building – D02, The University of Sydney NSW 2006, Australia; 2 School of Public Health, Room 4.14A, Building L05, Logan Campus, Griffith University, University Drive, Meadowbrook Queensland 4131, Australia

## Abstract

**Background:**

It is accepted knowledge that social and economic conditions – like education and income – affect population health. What remains uncertain is whether the degree of *inequality *in these conditions influences population health and if so, how. Some researchers who argue that inequalities are important, say there is a relationship between political economy, inequality and population health. Their evidence comes from comparative studies showing that countries with neo-liberal political economies generally have poorer population health outcomes than those with social or Christian democratic political economies. According to these researchers, neo-liberal political economies adopt labour market and welfare state policies that lead to greater levels of inequality and poorer population health outcomes for us all.

**Discussion:**

Australia has experienced considerable social and economic reforms over the last 20 years, with both major political parties increasingly adopting neo-liberal policies. Despite these reforms, population health outcomes are amongst the best in the world.

**Summary:**

Australia appears to contest theories suggesting a link between political economy and population health. To progress our understanding, researchers need to concentrate on policy areas outside health – such as welfare, economics and industrial relations. We need to do longitudinal studies on how reforms in these areas affect levels of social and economic inequality, as well population health. We need to draw on social scientific methods, especially concerning case selection, to advance our understanding of casual relationships in policy studies. It is important to find out if, and why, Australia has resisted the affects of neo-liberalism on population health so we ensure our high standards are maintained in the future.

## Background

Most population health researchers accept that social and economic conditions, such as such as levels of education, types of work and rates of unemployment, affect population health. Many suggest that the degree of inequality or disparity in social and economic conditions is important, and argue that inequality is a determinant of population health. They present evidence of a trend – countries with greater inequality tend to have poorer health outcomes overall as well as a more unequal distribution of health^1^. There is uncertainty however, about how the relationship between inequality and health works, that is, how the social is translated into the biological. Some researchers suggest that relative income inequality is the mechanism underlying the relationship. Wilkinson, for example, argued that those who experience relative deprivation suffer the psychosocial effects of chronic stress, which adversely affects their health [1,2]. When the experience of relative deprivation is widespread, it has a negative impact on overall population health outcomes. A more 'upstream' view is that politics, or more specifically, political economy is the principal cause of inequality [3-9]. Proponents of this view focus on the interdependence of the political and economic systems. They argue that income inequality is merely a symptom of the relationship between population health and inequality more generally [3]. Although they focus on different causes, these views are complimentary because relative inequality and political economy share the causal pathway linking the social and the biological.

Vicente Navarro has done the most work investigating the links between political economy and population health. Initially, he investigated the association between political economy type and socio-economic conditions in various countries. He found that, compared to social democratic or Christian democratic political economies, liberal political economies had higher income inequality and unemployment, lower wage and salary levels and a higher proportion of people living in poverty [3,5,10]. He attributed the worse conditions in liberal political economies to an erosion of the welfare state.

Navarro continued to research in the area, and collaborated with various colleagues to investigate the links between political economy and population health. Navarro and Shi [11] found that countries with liberal political economies had the largest income and wage differentials, the least redistributive impact of the state, and the lowest rate of improvement in infant mortality between 1960 and 1996. Navarro and the International Network on Social Inequalities and Health [6,7] tried to identify the reasons for this. They found that the labour market and welfare states policies of liberal political economies had a negative impact on social inequalities and mortality indicators.

## Discussion

Australia has not been considered by international researchers studying inequality and population health. Its political economy, along with other Anglo-Saxon countries like the United States (US) and the United Kingdom (UK), began to shift towards neo-liberalism in the 1980s [12]. During this period, there was an internationalisation of the economy, adoption of the managerial model and privatisation of the public sector, and industrial reforms that downgraded workers' entitlements. In the health care sector, we saw the increasing adoption of private sector principles and practices, an increased emphasis on transparency and accountability, and the introduction of managerial initiatives such as casemix-based hospital funding [13]. The period was also characterised by the removal of protection for the manufacturing and agriculture sectors, and a decline in unionisation [14]. The economic model adopted by the Hawke and Keating Labor Governments, and subsequently by the Howard coalition government, was 'characterised by a total reliance on, and faith in, market relations' [12]. While there were gains in productivity, efficiency and profitability, they came at a high social cost – greater inequality in wealth, income and social power [14].

Despite Australia's shift towards neo-liberalism and changes in social and economic conditions, population health outcomes remain amongst the best in the world [15]. This makes it an interesting case to consider. Table 1 shows where Australia ranks on various commonly used population health indicators. To allow comparisons, the country with the best population health outcome is also displayed.

**Table 1:** Population health indicators-Australia, rank and comparison to country with best outcome

**Population health indicator**	**Australia (Rank)**	**Country with best outcome (outcome)**
**Life expectancy at birth (years)**	79 (3^rd^)	Japan (82)
**Infant mortality rate per 1000**	6 (4^th^)	Japan, Singapore, Sweden (3)
**Under 5 mortality rate per 1000**	6 (4^th^)	Sweden (3)
**Adult mortality rate per 1000 (male)**	100 (6^th^)	Sweden (87)
**Adult mortality rate per 1000 (female)**	52 (5^th^)	Japan (44)
**Survival to age 65 (%) (male)**	84 (3^rd^)	Japan (86)
**Survival to age 65 (%) (female)**	92 (3^rd^)	Japan (94)
HALE at birth*	71.6 (4^th^)	Japan (73.6)

Sources: World Development Indicators 2004^15^, HALE = Healthy Life Expectancy^16^

Australia ranks third in the world on life expectancy at birth, third on survival to age 65 (male and female), fourth in IMR and under-five mortality rates, sixth on male adult mortality rate, fifth on female adult mortality rate and fourth on the HALE [16]. It is not possible to display data for other countries with liberal political economies in this paper, however when this data were examined, of all the countries with liberal political economies, Australia had the best population health indicators overall (excluding Japan) [17].

Japan ranks near, or at the top, for most population health indicators, even though it is also a liberal political economy. Japan's ranking may initially appear to contest the hypothesis that liberal political economies have poorer population health outcomes, casting doubt on its validity. However, it is important to consider that many features of Japan's political economy closely resemble those of social and Christian democratic political economies; it has a relatively equitable income distribution, high pension commitments and practises 'stakeholder' rather than 'shareholder' capitalism [18].

Although this is only a preliminary examination, Australian results also run counter to the hypothesis on the relationship between political economy, inequality and population health. To further examine this relationship, more complex and rigorous statistical studies are undoubtedly needed. If these studies prove that Australia does indeed defy the trend, there may be several reasons for this. First, Australia may be miss-classified as a neo-liberal political economy. Its political economy may be similar to those of the US and UK, but crucial differences that account for the variation in population health outcomes may not have been considered. One difference may be in the operation of the welfare state. The 'wage earners' welfare state model used in Australia has been long regarded as unique. It differs from the typical democratic socialist models, like that of the UK, but also from liberal models, like that of the US [19]. Australia's unique welfare state model may also partly account for its exceptionalism in terms of population health outcomes. Second, there may be a time-lag effect, where the shift towards neo-liberalism has yet to have an impact on population health outcomes. Third, there is the possibility that the hypothesis promoted by Navarro and others is limited, and when applied to a wider selection of countries, it is unable to explain the relationship between inequality and population health.

These contingent explanations for Australia's exceptionalism suggest some alternative directions for research in this area- both in terms of content and methodology.

As population health researchers, we need to expand our view and consider the variety of policy tools governments use to achieve their goals, not just those within the health sector. We should start by examining how labour market variables (such as unemployment and participation rates) and welfare state variables (such as public health care coverage and social protection expenditure) affect population health outcomes, as suggested by Navarro and the International Network on Social Inequalities and Health. However, we need to look more broadly than this and consider how changes in economic and industrial relations policies influence both the degree of inequality and population health outcomes in Australia. The Hawke government's Accord (where the unions agreed to exercise wage restraint in exchange for the 'social wage' benefits of Medicare [20]) is an example of how important it is to examine the integral links between industrial relations, economic and health policies when evaluating the association between political economy and population health in Australia.

Prospective as well as retrospective longitudinal studies need to be conducted because of the possible time-lag between policy reform and tangible evidence of change. Australia's exceptionalism implies that international comparative studies be undertaken, but special attention should be given to selection of case studies for comparison. Cases must be selected on the basis that they are likely to be help uncover the underlying causal relationships between policy, inequality and health. They should not be selected merely because they are convenient or conventional comparisons. The social science literature offers much to health policy researchers interested in policy study methodology and provides a useful starting point for ongoing research in the area.^2^

## Summary

Australia's apparent challenge to current theories on the relationship between political economy type and population health outcomes needs to be explored in more detail. Further research needs to include investigations into the impact of labour market, welfare state, economic and industrial relations policies on inequality and population health outcomes. They should observe changes in conditions over time and make use of the existing knowledge within the social sciences on case study methodology. These questions are worth considering because the link between inequality and population health is important, but remains unclear. It is important that Australian policy makers and researchers make these investigations a priority as relying on our exceptionalism may prove to be a high-risk approach to protecting the nation's health.

## Competing interests

The author(s) declare that they have no competing interests.

## Authors' contributions

AB carried out the study, upon which this manuscript is based and prepared the manuscript draft. SS contributed to the development of ideas expressed in the manuscript and helped prepare the manuscript draft. Both authors read and approved the final manuscript.

## Appendix

^1 ^See for example Lynch J, Davey Smith G, Hillemeir M, Shaw M, Ragunathan T and Kaplan G (2001). Income inequality, the psychosocial environment, and health: comparisons of wealthy nations. The Lancet 358 (9277) (21 July): 194–200; Navarro V. (1998). Neo-liberalism, "globalisation", unemployment, inequalities, and the welfare state. International Journal of Health Services 28(4): 607–682 and Navarro V. (1999b). The political economy of the welfare state in developed capitalist countries. International Journal of Health Services 29 (1): 1–50.

^2 ^See for example King G, Keohane, RO and Verba S. (1994). Designing Social Inquiry: Scientific Inference in Qualitative Research. Princeton, New Jersey, Princeton University Press; and George AL and Bennett A (2005). Case Studies and Theory Development in the Social Sciences. Cambridge, Massachusetts, MIT Press.
